# Advancing Objective Mobile Device Use Measurement in Children Ages 6–11 Through Built-In Device Sensors: A Proof-of-Concept Study

**DOI:** 10.1155/2024/5860114

**Published:** 2024-05-28

**Authors:** Olivia L. Finnegan, R. Glenn Weaver, Hongpeng Yang, James W. White, Srihari Nelakuditi, Zifei Zhong, Rahul Ghosal, Yan Tong, Aliye B. Cepni, Elizabeth L. Adams, Sarah Burkart, Michael W. Beets, Bridget Armstrong

**Affiliations:** 1 Department of Exercise Science, University of South Carolina, Columbia, South Carolina, USA; 2 Department of Computer Science and Engineering, University of South Carolina, Columbia, South Carolina, USA; 3 Department of Epidemiology and Biostatistics, University of South Carolina, Columbia, South Carolina, USA

**Keywords:** digital media use, mobile screen use, objective measurement

## Abstract

Mobile devices (e.g., tablets and smartphones) have been rapidly integrated into the lives of children and have impacted how children engage with digital media. The portability of these devices allows for sporadic, on-demand interaction, reducing the accuracy of self-report estimates of mobile device use. Passive sensing applications objectively monitor time spent on a given device but are unable to identify who is using the device, a significant limitation in child screen time research. Behavioral biometric authentication, using embedded mobile device sensors to continuously authenticate users, could be applied to address this limitation. This study examined the preliminary accuracy of machine learning models trained on iPad sensor data to identify the unique user of the device in a sample of children ages 6 to 11. Data was collected opportunistically from nine participants (8.2 ± 1.75 years, 5 female) in the sedentary portion of two semistructured physical activity protocols. SensorLog was downloaded onto study iPads and collected data from the accelerometer, gyroscope, and magnetometer sensors while the participant interacted with the iPad. Five machine learning models, logistic regression (LR), support vector machine, neural net (NN), k-nearest neighbors (k-NN), and random forest (RF), were trained using 57 features generated from the sensor output to perform multiclass classification. A train-test split of 80%–20% was used for model fitting. Model performance was evaluated using *F*1 score, accuracy, precision, and recall. Model performance was high, with *F*1 scores ranging from 0.75 to 0.94. RF and k-NN had the highest performance across metrics, with *F*1 scores of 0.94 for both models. This study highlights the potential of using existing mobile device sensors to continuously identify the user of a device in the context of screen time measurement. Future research should explore the performance of this technology in larger samples of children and in free-living environments.

## 1. Introduction

Mobile devices (e.g., tablets and smartphones) have become ubiquitous in the lives of children, with 78% of children under the age of 8 having access to a tablet in their home [1]. Tablets are favored by children, primarily because of their interactive features, visual appeal, and access to a wide range of media [2]. As children are spending increasingly more time engaging with mobile devices, concerns have been raised about the long-term implications of mobile device use for health and developmental outcomes [3–6]. These concerns have initiated research efforts to improve current measures of mobile device use for the goal of better understanding these links with health outcomes [7].

The introduction of mobile devices has substantially impacted the ways in which children consume media, such that children are more apt to use these devices sporadically due to the portable nature of these devices [8–10]. Historically, screen time has most commonly been measured through retrospective self-report or proxy-report [9, 10]; however, the introduction of on-demand and portable devices warrants updated measures of screen time. Given that high-frequency behaviors, such as checking a mobile device, are challenging to report retrospectively [11], there is a demand for objective measures that more proximally capture mobile device use.

Passive sensing applications have been introduced as a method to objectively monitor time spent on a specific device [10]. Chronicle, an Android passive sensing application, tracks a variety of metrics, including the duration, frequency, timing, general application type, and application status, by interfacing with Google API every 15 seconds. While self-report methods are not sensitive enough to fully capture all mobile device use, passive sensing applications can address this limitation by producing highly reliable data [8]. However, the critical limitation inherent to using passive sensing applications is that they do not provide an indication of who is interacting with the device at specific time points. While this is of less concern in adult screen time research, this limitation is critical in child screen time research where mobile devices are often shared across individuals within a family [8]. In order to enhance the validity of passive sensing applications for children, it is necessary to continuously track who is using the device, in conjunction with the metrics provided by passive sensing applications (e.g., duration, frequency, and timing).

A potential solution to address this limitation is leveraging behavioral biometric authentication, an established field of research within the field of cybersecurity. Behavioral biometric authentication refers to using built-in mobile device sensors (e.g., accelerometer and gyroscope) to continuously authenticate users through machine learning models [12]. This technology exists on the premise that different individuals have distinct movement patterns in the ways that they interact with mobile devices [13]. Features derived from the sensor output are fed into machine learning models and then authenticate users of a device over a selected window of time [14]. This field in cybersecurity has grown in recent years in an effort to improve security on devices [15]; however, this technology has not yet been applied to measurement of children’s mobile device use, which has the potential to improve the accuracy of passive sensing and thereby fill a critical public health need.

A necessary first step toward using biometric authentication technology to measure device use in pediatric populations is to train models using mobile device sensor data collected in a sample of children. Therefore, the objective of this study was to estimate the preliminary predictive accuracy of machine learning approaches trained on iPad sensor data (accelerometer, gyroscope) to identify a unique user in a sample of children ages 6 to 11.

## 2. Methods

### 2.1. Sample.

Data for the current study was collected opportunistically from two existing semistructured physical activity study protocols, PATCH and Wearables for Kids (W4K). The exclusion criteria of the first study, PATCH, included the diagnosis of autism, pervasive development disorder, or contraindications to exercise (e.g., orthopedic injuries and heart conditions). Children were recruited through Facebook ads, the University of South Carolina newsletter, and referrals. The inclusion criteria of the second study, W4K, were the ability to be physically active without an assistive device, such as a wheelchair, while exclusion criteria included the diagnosis of a condition known to affect heart rate (HR), a neuromuscular disease, and/or the prescription of medications known to affect HR. Children were recruited through after-school programs and summer day camps of the greater Columbia, South Carolina area, as well as through newsletters, social media, and referrals.

For both PATCH and W4K, interested parents were directed to complete an online survey to determine eligibility and complete an online consent form. Consent was then confirmed with the parent over the phone, and child assent was provided directly before the protocol once the child arrived. Families received a $50 gift card for participation in PATCH and a $40 gift card for participation in W4K upon completion of the study.

Nine participants, with 5 from the W4K study and 4 from the PATCH study, were included in this study. The sample size of nine participants is justified for several reasons: (1) As this is a preliminary proof-of-concept study, this sample size was selected to demonstrate the *initial potential* of applying this technology to the screen time domain. (2) This sample size is consistent with the literature in the field of biometric authentication. Within the field of biometric authentication, smaller sample sizes have been used for the preliminary testing of model performance in authenticating device users (e.g., 5–10 participants) [16–20]. (3) The machine learning models used in this study are trained to predict the child user at the 1-s level (i.e., with a high degree of granularity); therefore, the amount of data points per subject is large (see Table S4). (4) The principle of cross-validation was applied in the machine learning model development, such that models were trained on 80% of an individual’s data and tested on a different 20% of an individual’s data, thereby increasing the robustness of the models to detect users even with a relatively small sample size.

### 2.2. Protocol Description.

The PATCH study physical activity protocol, described in full detail elsewhere [21], took place at the University of South Carolina, with the opportunistic data of this study being collected between October 2022 and December 2022. This 45-min lab-based physical activity protocol was designed to have children engage in activities ranging from sedentary to vigorous physical activity to simulate free-living movement. The current study leveraged the sedentary portion of the PATCH protocol, in which children were interacting with iPads. As part of the protocol (Table S2), there were six separate sedentary periods (for a total of 35 min), in which children self-selected games (e.g., Candy Crush Saga, Subway Surfers, and Pet Doctor) or videos (e.g., PBS KIDS Video and YouTube Kids) on iPads.

The W4K study physical activity protocol, described in full detail elsewhere [22], took place at after-school programs, summer day camps, and the Public Health Research Center at the University of South Carolina. The opportunistic data of this study was collected between February and March 2023. Children were equipped with a Cosmed K5 portable calorimeter, an ActiHeart monitor, a research-grade accelerometer (ActiGraph GT9X), and two consumer wearables (Garmin vivoactive, Apple Watch Series 7, and Fitbit Sense). Similar to PATCH, the semistructured protocol was designed to engage children in activities across all intensity levels (sedentary to vigorous, see Table S1). The rest period (10 min) and sedentary activity (5 min) of the W4K protocol were leveraged for the current project and totaled 15 min. For the first sedentary activity, children laid down and were instructed to either self-select a video to watch or a game to play on the iPad for the 10-min rest period. In the second sedentary activity, children self-selected a video to watch or a game to play while seated for 5 min.

For both the PATCH and W4K protocols, research assistants recorded the start and end time of each activity of the protocol to the 1-s level. Research assistants directly observed participant use of the iPad and logged time on the iPad to the second level.

### 2.3. Sensor Tracking Technology and Sensors Selected.

**Figure 1:**
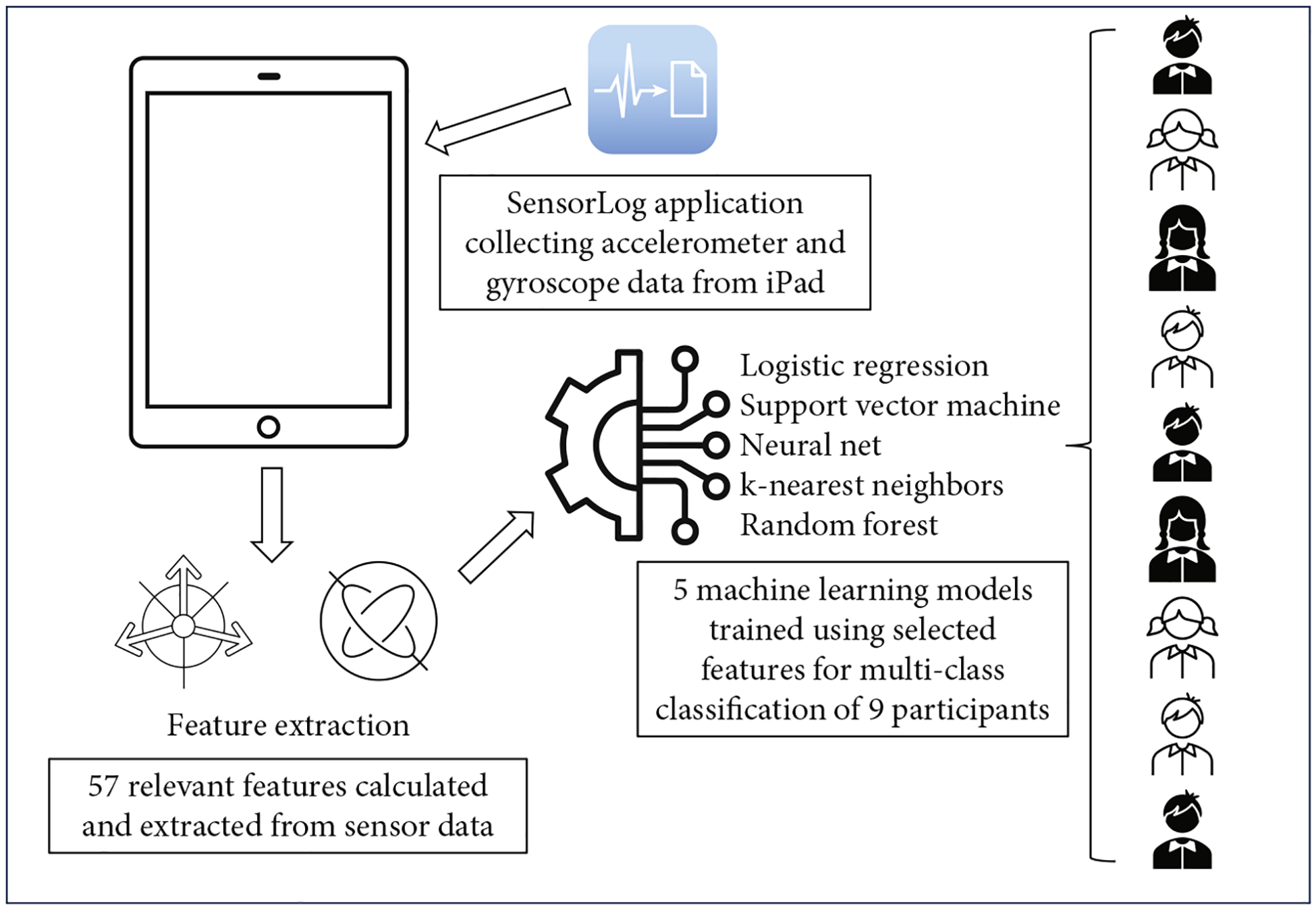
Graphic abstract of study methodology.

Figure 1 presents a graphic abstract of the study methodology. Prior to the start of each W4K and PATCH protocol, trained research assistants activated the iOS application SensorLog (https://apps.apple.com/us/app/sensorlog/id388014573). SensorLog is an open-source application that leverages device APIs to continuously log sensor data from iOS devices at up to 100 Hz. This application can access a variety of built-in sensors and data streams on mobile devices, including coordinates, speed, altitude, accelerometer, gyroscope, magnetometer, gravity, rotation, steps, distance, pace, cadence, and battery. The SensorLog application interface for the accelerometer sensor is presented in Figure S2. The current study only gathered data from the accelerometer, gyroscope, and magnetometer sensors built into the iPad devices. The accelerometer measures acceleration, which is the rate of change of the velocity of the device, along three axes. The gyroscope measures angular velocity, which is the rate of rotation around the three axes. The magnetometer measures the strength of the Earth’s magnetic field in reference to the three axes of the mobile device. Both the accelerometer and gyroscope have been widely used in research on user authentication and identification [23–25].

For all PATCH participants, the sampling frequency was set to 20 Hz. For 3 of the W4K participants, the sampling frequency was set to 100 Hz, while for the other two W4K participants, the frequency was set to 20 Hz. Within the field of biometric authentication, researchers have used sampling frequencies from 1 to 100 Hz; therefore, the sampling frequencies of 20 and 100 Hz are sufficient to capture movement related to device usage [26–31]. The application was set to record raw accelerometer data (*X*, *Y*, and *Z* axes) in gravity, raw gyroscope data (*X*, *Y*, and *Z* axes) in radians/second, and raw magnetometer data (*X*, *Y*, and *Z* axes) in Ậ*μT* units. On the SensorLog settings, the current study selected to record unbiased user acceleration, altitude, gravity, heading, magnetic field, and rotation, which provided motion yaw, pitch, roll in radians, and motion quaternions in the *X*, *Y*, and *Z* axes in R units.

### 2.4. Characteristics of Devices Evaluated.

iPads (ninth generation with iOS 16.5.1) were selected for the following reasons: (1) They have built-in sensors that capture acceleration, motion, and orientation. (2) Tablets like the iPad are more likely to be shared across siblings or within the family. (3) This same technology could be applied in mobile devices such as iPhones, as the SensorLog iOS application is available across iOS devices.

### 2.5. Data Preprocessing and Processing.

Following the protocol, data were exported as a CSV which was then input to a Python script for feature extraction and analysis. After data were exported, the number of samples per time window (1 s) was assessed to ensure full data coverage. A time window was removed if data samples within one second were less than a quarter of the sampling rate (< 25 samples per second (100 Hz), < 5 samples per second (20 Hz)). Eleven time windows were removed due to insufficient data coverage. This represents 0.00096% of the data. Consistent with previous studies of user authentication [25, 32–34], data were mean aggregated to the 1-s level, and feature vectors were calculated from each time window.

After data cleaning and preprocessing, a min–max normalization technique was applied, which maps the feature values into the range in [0,1] based on the min and max of features. This technique is commonly done with analyses in biometric authentication [35, 36] and particularly in analyses using k-nearest neighbors (k-NN) machine learning model [37, 38], as it has better performance than other normalization techniques, such as *z*-score normalization [39]. A scikit-learn package was used to perform this normalization technique (https://scikit-learn.org/stable/modules/generated/sklearn.preprocessing.MinMaxScaler.html) (Python v. 3.8, Delaware, United States).

### 2.6. Feature Extraction and Selection.

The goal of feature selection in this study was to identify features that were discriminating with respect to the way that the user interacts with the device [14]. The limited biometric authentication research in children has used features including the mean, standard deviation, variance, minimum, maximum, root-mean-square-deviation, skewness, and kurtosis of each data stream (i.e., acceleration in *X*, *Y*, and *Z* axes), with high performance in classifying the user [33]. Additionally, previous biometric authentication research in child populations has used yaw, pitch, and roll (average, standard deviation, average deviation, root mean square, and minimum and maximum), with high authentication performance particularly in maximum yaw, average yaw, and minimum yaw [40]. Pitch, yaw, and roll are the three dimensions of movements, or rotational forces, about the *X*, *Y*, and *Z* axes, generated by the accelerometer and gyroscope. Pitch is the rotation about the *X* axis, yaw is the rotation about the *Y* axis, and roll is the rotation about the *Z* axis. To calculate pitch, yaw, and roll, quarternions first had to be calculated. Although the SensorLog application output provides estimates for pitch, yaw, and roll, Apple does not provide documentation for how they are calculated. Therefore, to maximize reproducibility and transparency, these metrics were calculated using freely available software (https://gist.github.com/phausamann/721fa3df0f8ef6f4f6f24b86fdde53c0). The calculated pitch, yaw, and roll (mean, standard deviation, variance, minimum, maximum, root-mean-square deviation, skewness, and kurtosis) from the current study were used for the full analyses. In line with previous biometric authentication research, the full feature set of this study additionally included the mean, standard deviation, variance, minimum, maximum, root-mean-square deviation, skewness, and kurtosis of each data stream. In total, 57 features were used to train the models (see Table S3).

### 2.7. Model Training and Validation.

The data was divided into two parts: 80% for model training and 20% for model testing, consistent with previous research [41]. The number of data samples used for training and testing for each participant is displayed in Table S4. Multiclass classification was performed, which classifies each test sample into more than two classes, in this case nine classes. In this analysis, the multiclass model classifies at each second whether the user was child A or child B or child C (of all nine participants).

### 2.8. Machine Learning Models.

Machine learning is a branch of artificial intelligence that focuses on developing algorithms capable of automatically improving their performance through experience. By analyzing large amounts of data, these algorithms identify patterns and relationships, allowing them to make predictions or decisions without explicit programming. Five popular machine learning models, logistic regression (LR), support vector machine (SVM), neural net (NN), k-NN, and random forest (RF), were used in the current study. LR functions by predicting the odds of an outcome [42]. LR has been used as a classifier with sensor-based methods and works effectively in predicting the probability of different classes, making it a reasonable selection for the current analysis [33, 43]. k-NN is a supervised machine learning algorithm that uses nearby data points to make predictions [44], and SVM is a supervised machine learning algorithm that maps a line (hyperplane) to optimize the distance between different classes [45]. k-NN offers simplicity and speed without assumptions about data (e.g., distribution of the data) [44], while SVM excels in classification by finding an optimal separating hyperplane, even by using kernels like the radial basis function (RBF). SVM functions by mapping data to a high-dimensional feature space and has been applied to classification within the field of biometric authentication [33, 46–48]. k-NN has also been largely applied to biometric authentication [31, 49–53], given its strength in the classification of data where the distribution is not normally distributed [44]. RF is a popular supervised machine learning algorithm that uses branched decision-making through trees. More specifically, RF functions by fitting several decision tree classifiers on subsamples of the dataset and then averaging to optimize predictive accuracy and reduce overfitting [54]. NN is a feed-forward neural network that is made up of interconnected nodes that are arranged in layers and is largely used for multiclass classification problems [55]. RF and NN have both been used widely in adult biometric authentication research using mobile device sensors [49, 52, 56–60].

Hyperparameters are commonly used in machine learning as external configuration variables (e.g., nodes and layers) to help in controlling the machine learning model training [61]. For the selection of hyperparameters, grid search was applied with 3-fold cross-validation using the training dataset to choose the best parameters. The following parameter ranges were set: parameter *k* = {5,10,100,200} for k-NN, the complexity parameters *c* = {2^−5^, 2^−4^, .., 2^4^, 2^5^} for SVM and LR, the maximum depth of the tree *d* = {10,50,100} for RF, and the strength of the L2 regularization term *α* = {10^−1^, 10^−2^, ⋯, 10^−5^} for NN. The final best parameters determined by grid search are *k* = 10, *c* = 2^4^, *d* = 100, and *α* = 10^−4^.

### 2.9. Model Evaluation Metrics.

The performance of the models was evaluated using popular metrics: *F*1 score, accuracy, precision, and recall, for each participant following a multiclass strategy. For all metrics, possible scores range from 0 to 1, and values closer to 1 are better. The *F*1 score combines the precision and recall scores of a model into a weighted score. Accuracy is a common and straightforward metric that measures the overall correctness of predictions made by a classification model ((true positives + true negatives)/all observations). Precision measures the positive predictive ability of a model (true positives/(true positives + false positives)). Recall measures the ability of the model to correctly identify all positive instances (true positives/(true positives + false negatives)). The overall model performance was evaluated as the average of the nine participants. The goal in selecting the optimal model was to maximize *F*1 score, accuracy, precision, and recall. Lastly, confusion matrices were also visually inspected to evaluate the performance of the multiclass classifier, as these matrices compare predicted observations to the ground truth. These confusion matrices were additionally used to understand trends of misclassification in the models [62].

### 2.10. Feature Importance.

Feature importance was evaluated of all included features in the RF model using the Gini importance. This impurity-based method for feature ranking computes the importance of features by calculating the normalized total reduction of the criterion introduced by that feature [63]. Higher values on the Gini importance scale indicate better performance, and the total importance of all features sums to one.

## 3. Results

Demographic characteristics of the sample are presented in Table 1. Given the similarity between the two semistructured protocols and since this study leveraged data only from the sedentary portion of those protocols, participants from the PATCH protocol and the W4K protocol were analyzed together. As a sensitivity analysis, the difference in performance when analyzed separately was assessed (see Figure S1). The estimates produced when modeled with the participants collapsed into one dataset versus analyzed separately were similar, justifying the decision to analyze all nine participants together.

### 3.1. Model Performance.

The evaluation metric performance for all machine learning algorithms is presented in Table 2, showing the overall multiclass performance across all nine participants. The best performing models were the RF and k-NN classifiers, yielding overall classification accuracies of 0.94 (precision = 0.94, recall = 0.94). NN also was high performing across all evaluation metrics, producing an *F*1 score of 0.92. The lowest performing algorithms were SVM and LR, with *F*1 scores of 0.85 and 0.75, respectively.

**Figure 2:**
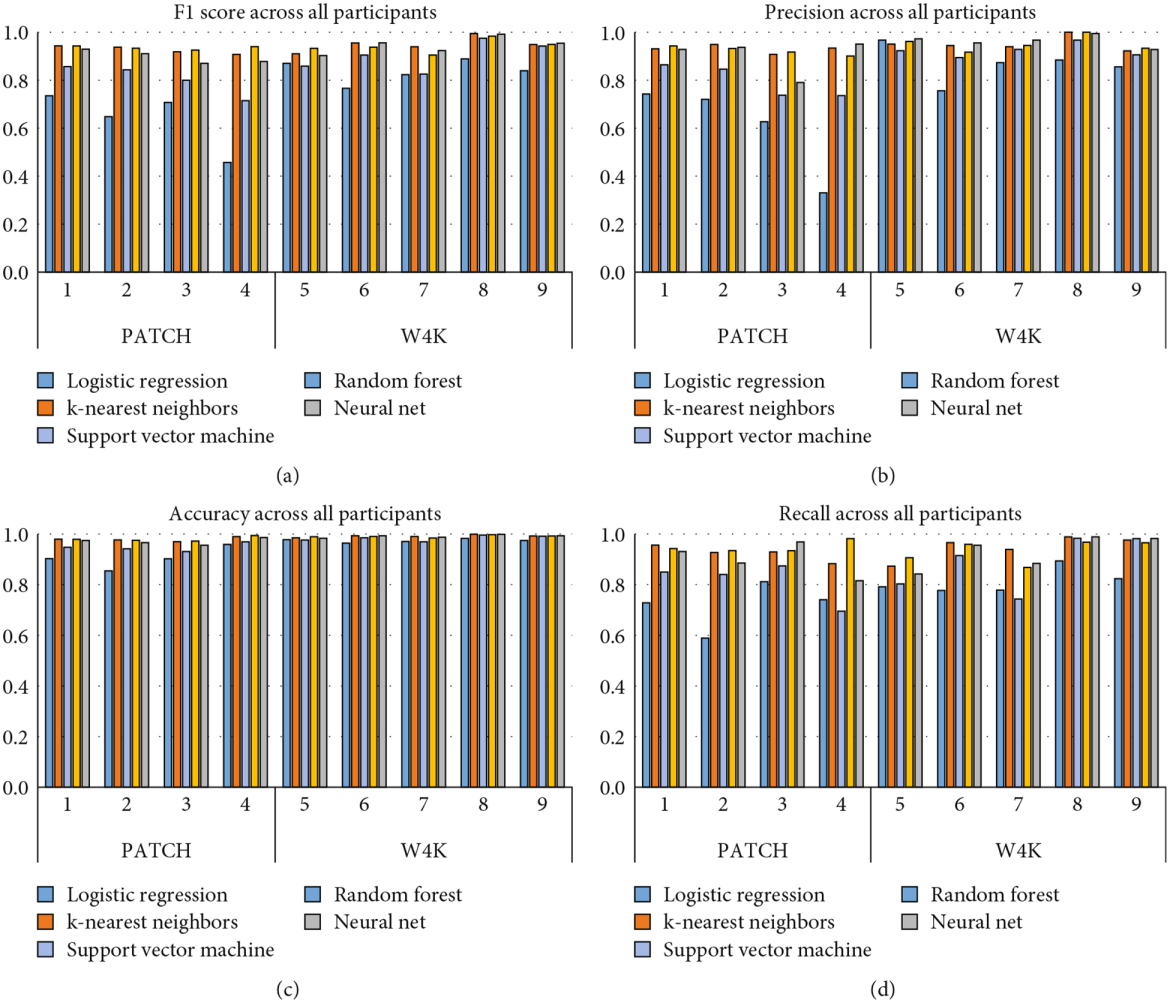
(a–d) Performance evaluation metrics by participant.

Classifier performance by participant is displayed in Figures 2(a)–2(d).*F*1 score results are shown in Figure 2(a), where the highest *F*1 score was achieved with both the k-NN model and the NN algorithm on Participant 8 with a score of 0.99. Accuracy results are displayed in Figure 2(b). The best performing model when assessing accuracy was achieved by k-NN in Participant 8 with a score of 0.99. The worst performing model in terms of accuracy was LR in Participant 2 with a score of 0.85. Figure 2(c) displays precision results, with the best performing models achieved with k-NN in Participant 8 and NN in Participant 8 with a precision of one. A precision of one indicates that the model has no false positives, meaning that it is never incorrectly identifying Participant 8 when it is not Participant 8. The lowest precision was found in the LR model for Participant 4, with a precision of 0.33. Recall results are presented in Figure 2(d). The best performing models in terms of recall were k-NN for Participant 8 with a recall of 0.99 and NN for Participant 8 with a recall of 0.99. The worst performing models in terms of recall were LR in Participant 2 with a recall of 0.59 and LR in Participant 1 with a recall of 0.73.

**Figure 3:**
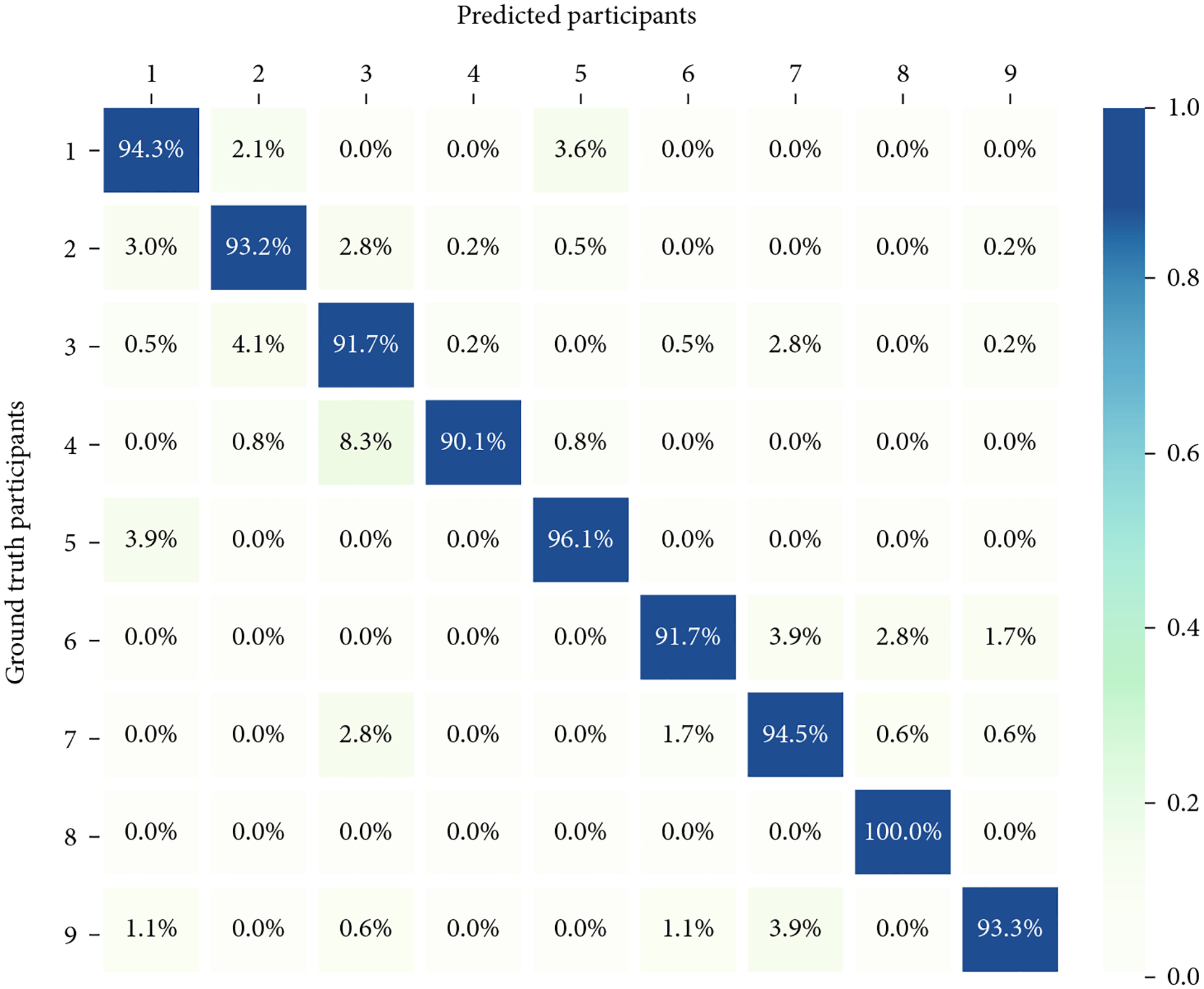
Confusion matrix of highest performing model (random forest).

A heat map confusion matrix representing the multiclass classification results for RF is displayed in Figure 3. This confusion matrix displays the predicted user compared to the ground truth (the actual user of the iPad). In assessing this confusion matrix, classification agreement was very high across all participants (> 0.90), indicating excellent performance. For Participant 8, the model correctly identified the user against all participants, producing an accuracy of one.

### 3.2. Feature Importance.

**Figure 4:**
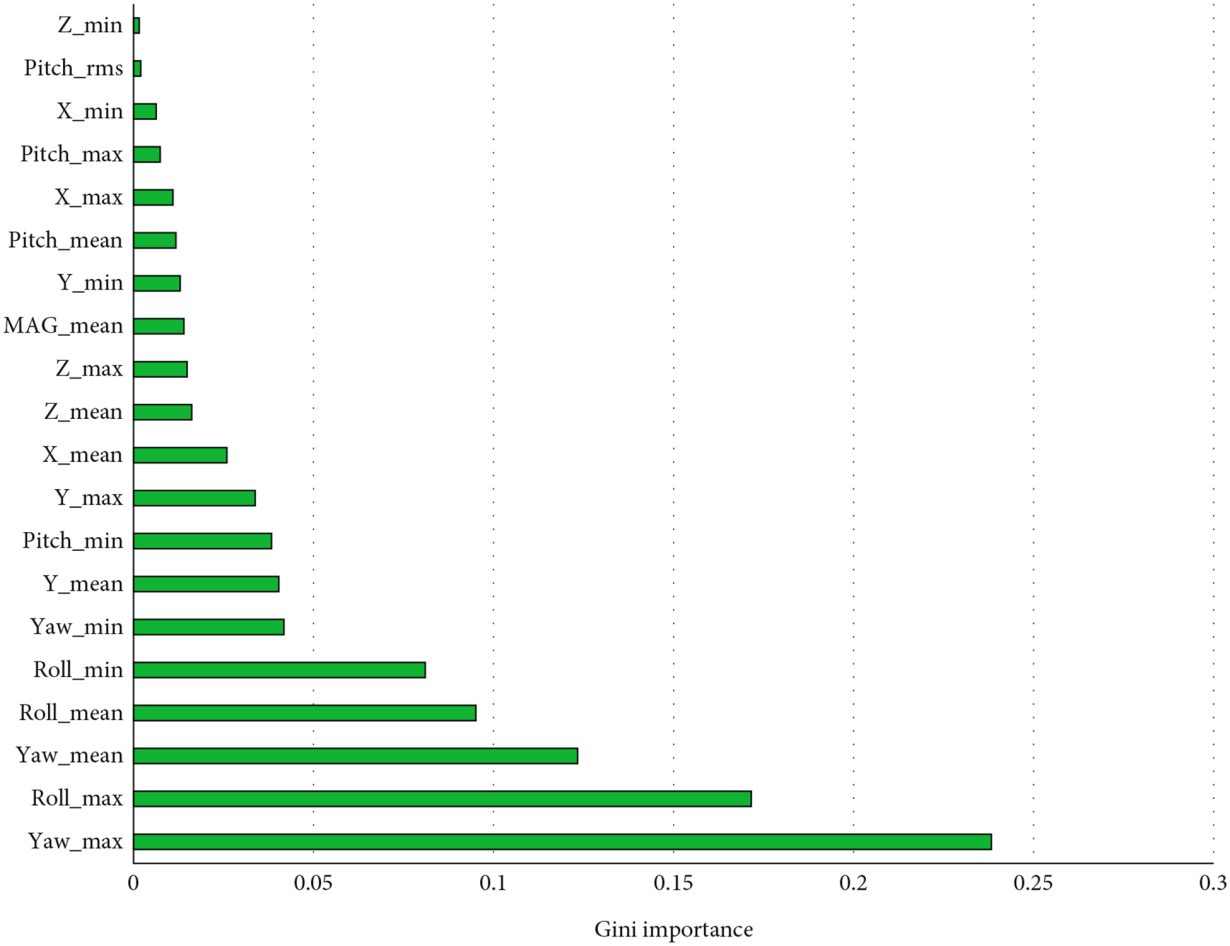
Ranking of top 20 features in highest performing model (random forest). Higher values on the Gini importance scale indicate stronger contribution of that feature to the model performance. Abbreviations: MAG_mean: mean vector magnitude, Pitch_max: maximum pitch, Pitch_mean: mean pitch, Pitch_min: minimum pitch, Pitch_rms: root mean square of pitch, Roll_max: maximum roll, Roll_mean: mean roll, Roll_min: minimum roll, *X*_max: maximum acceleration along the *X* axis, *X*_mean: mean acceleration along the *X* axis, *X*_min: minimum acceleration along the *X* axis, *Y*_max: maximum acceleration along the *Y* axis, *Y*_mean: mean acceleration along *Y* axis, *Y*_min: minimum acceleration along the *Y* axis, Yaw_max: maximum yaw, Yaw_mean: mean yaw, Yaw_min: minimum yaw, *Z*_max: maximum acceleration along the *Z* axis, *Z*_mean: mean acceleration along the *Z* axis, *Z*_min: minimum acceleration along the *Z* axis.

The ranking of feature importance using the Gini importance based on the RF model is displayed in Figure 4. The highest performing features were maximum yaw, maximum roll, mean yaw, mean roll, and minimum roll, with all five of these features each contributing over 0.05 in feature importance. The maximum yaw feature was the best performing at 0.24. Maximum roll had a feature importance of 0.17, mean yaw had a feature importance of 0.12, and mean roll had a feature importance of 0.10. The features that performed most poorly were minimum acceleration on the *X* and *Z* axes, root-mean-square of pitch, and maximum pitch.

## 4. Discussion

The purpose of this proof-of-concept study was to test if machine learning models trained on mobile device sensor data could identify a unique user in a sample of children ages 6 to 11. This study demonstrated the initial potential of applying biometric authentication to overcome the limitations of objective mobile device use measurement. This was done through data collected opportunistically during two semistructured physical activity protocols. Using biometric authentication in conjunction with passive sensing applications (e.g., Chronicle) has the potential to improve the accuracy of current estimates of mobile device use in children to better understand the interdependent relationship between mobile device use and health outcomes.

The findings of this study highlight the strength of these models trained on iPad accelerometer and gyroscope sensor data in multiclass classification to identify a unique user among nine participants. All evaluation metrics showed that RF, k-NN, and NN models were good at classifying what user was on the device. SVM and LR model performance was lower, although it still performed moderately well across all evaluation metrics.

While model performance was generally strong in this preliminary study, there was variability in performance among models. Both k-NN and RF performed comparably, with the same estimates across all evaluation metrics when averaged across all nine participants. The strength of the RF model is consistent with the literature in adult user identification [50–52, 64, 65]. Indeed, RF consistently outperforms other models [31, 50–52, 64–66], achieving an *F*1 score as high as 99.7% [64] in one study that used accelerometer, gyroscope, and magnetometer sensors for user identification in adults. Within the biometric authentication literature, while k-NN also produces high evaluation metrics, it does not *consistently* outperform other models [31, 49, 52]. One study using the accelerometer and gyroscope sensors from Android smartphones found that SVM and NN actually perform better than in k-NN [49]. However, SVM and NN models only performed marginally better, achieving accuracies of 96.3% and 91.4%, respectively, compared to the k-NN accuracy of 86.3%. These findings and other literature support the idea that future work using this technology in a similar context should opt to use the RF or k-NN models. Future work is also necessary to identify features and fine-tune models in a way that is optimal for distinguishing between users with similar behavior patterns. A potential avenue for this line of work is to use long short-term memory (LSTM), a machine learning approach that can handle sequential data, as it learns long-term dependencies across data [67]. This would be useful for user authentication because it is likely that the same child would be using a mobile device over a given bout of time and training the model with behavior from earlier in the bout would enhance model performance. However, LSTM models are computationally intensive [68], which is why more parsimonious techniques were used in this preliminary study.

The best performing features of the RF model in the current study were maximum yaw, maximum roll, and mean yaw. All of these features are derived from quaternions. Yaw and roll are the rotation about the *Y* axis and *Z* axis, respectively, which is measured through the accelerometer and gyroscope sensors. These same features have been used to identify child users through touchscreen gestures and hand stability measured through accelerometer and gyroscope sensors [40]. Interestingly, our findings along with previous research seem to highlight the importance of features derived from movement about the *Y* axis. A key distinction between both previous studies that used features derived from *Y* axis movement is that they aimed to identify demographic characteristics (i.e., detect whether the user is a child or an adult) [33, 40] whereas the current study is aimed at identifying the unique user in a sample of children. Of the hand stability features included in a similar study, maximum yaw, average yaw, and minimum yaw were the three most important features. Similarly, another study that sought to identify child users of a mobile device using accelerometer, gyroscope, linear acceleration, and rotation sensors found that maximum rotation around the *Y* axis, minimum rotation around the *Y* axis, and mean rotation around the *Y* axis were the three highest performing features [33].Although those studies aimed to identify demographic characteristics (i.e., age group), vertical axis movement appears to be a key discriminating feature that defines how a child interacts with a device.

Identifying the user of a given device is a critical need in the field of public health research. The current literature evaluating the impact of mobile devices on children’s health is limited by its reliance on parent-report measures [69]. While passive sensing applications like Chronicle present a viable way to objectively monitor the *content* and *timing* of mobile device use, critically, this method cannot distinguish who is actually using the device. This is a problem because estimates show roughly 66% of families share devices [8]. The current study represents a necessary first step in remedying this limitation, by applying existing biometric authentication technology to a new context, namely, children’s screen time measurement. This preliminary study has demonstrated the potential of using this technology (in conjunction with passive sensing applications) to gather more accurate estimates of screen time. Specifically, we found that motion sensors built into mobile devices can be harnessed to distinguish between users, which indicates that these methods are a promising avenue to further investigate in a larger and more diverse sample.

The limitations of this study should also be considered when interpreting these findings. While integrating data collection into existing semistructured physical activity protocols allowed for opportunistic data to be captured, there are inherent limitations in this study design. First, there may be differences between the W4K and PATCH protocol that would impact the way in which the child interacted with the device. However, these semistructured physical activity protocols were run simultaneously within the same university laboratory under very similar conditions. Also, as a sensitivity analysis, the W4K participants and PATCH participants were analyzed separately and the difference between the full analysis for the nine participants and the by-protocol analysis was negligible (see Figure S1). Therefore, it is unlikely that differences in protocols significantly impacted the results. Second, the game or video being played/watched during the semistructured physical activity protocols was not standardized. It is possible that the models are only identifying movements consistent with playing/watching a specific game/video and not the distinct movement differences between each participant. The specific game the child was watching or whether they were watching a video was not recorded; therefore, model performance cannot be compared across the types of activity. However, children chose from a specific selection of games (Candy Crush, Subway Surfers, etc.), and it is likely that more than one child played the same game. The variability in what children were doing (playing a game, watching a video) is more representative of how children would interact with the devices in a real-world setting. Nevertheless, future research should examine the strength of models in user identification when the game or video is standardized. Third, two different sampling frequencies were used to collect sensor data from the iPads, with six participants using an iPad sampling at 20 Hz and three participants using an iPad sampling at 100 Hz. However, data were aggregated to the 1-s level; therefore, this discrepancy in sampling frequency is unlikely to impact model performance. Fourth, it is possible that the models distinguished users based solely on the orientation of the iPad when the participant was holding it. Future studies can address this concern through simulated free-living protocols in which participants interact with the device for longer durations over multiple time periods. Lastly, given the preliminary nature of this study, data was only collected from nine participants. This small sample is in line with similar preliminary studies and sets the stage for future research with a larger sample to provide a more generalizable and realistic representation of how this can be deployed on a larger scale. Children may interact with mobile devices differently in free-living contexts; therefore, subsequent studies should also examine the performance of this technology in a free-living application.

## 5. Conclusions

In sum, these findings highlight the potential of leveraging built-in iPad sensors for user identification in children, with promising preliminary results in a sample of nine children ages 6 to 11. User identification through built-in mobile device sensors, when combined with passive sensing applications, has the potential to improve the objective assessment of children’s mobile device use. Mobile devices have quickly changed the ways in which children interact with and engage with digital media, and it is necessary to accurately measure screen time in order to inform public health recommendations. Future research should aim to replicate these results in a larger sample of children in both standardized lab-based and free-living conditions.

## Supplementary Material

Supplementary tables and figures

Additional supporting information can be found online in the Supporting Information section. Tables S1 and S2 The full semistructured physical activity protocols leveraged for this study (Wearables for Kids and the Patch Study, respectively). Figure S1 Confusion matrices when the random forest models were run by protocol (W4K, Patch). Table S3 All 57 features used in the current study. Table S4 The amount of training and testing data by participant, in number of seconds. Figure S2 A picture of the SensorLog application interface, which was the sensor tracking application used in this study. This picture shows the reading for the accelerometer sensor. (Supporting Information)

## Figures and Tables

**Table 1:** Demographics of the participating children (*n* = 9).

	W4K	PATCH
Sex	*n*	*n*
Female	4	1
Male	1	3
Race		
Black	2	0
White	3	4
Ethnicity		
Not Hispanic or Latinx	5	4
Hispanic or Latinx	0	0
	Mean (SD)	Mean (SD)
Age	9.2 (1.72)	7 (0.71)

Abbreviations: SD, standard deviation; W4K, Wearables for Kids.

**Table 2:** Performance evaluation metrics averaged across all participants (*n* = 9).

	F1 score	Accuracy	Precision	Recall
Logistic regression (LR)	0.75 (0.13)	0.74 (0.04)	0.77 (0.18)	0.74 (0.08)
Support vector machine (SVM)	0.85 (0.07)	0.85 (0.02)	0.86 (0.08)	0.85 (0.09)
Neural net (NN)	0.92 (0.04)	0.92 (0.01)	0.92 (0.06)	0.92 (0.06)
k-nearest neighbors (k-NN)	0.94 (0.03)	0.94 (0.01)	0.94 (0.02)	0.94 (0.04)
Random forest (RF)	0.94 (0.02)	0.94 (0.01)	0.94 (0.03)	0.94 (0.03)

*Note:* Presented as evaluation metric (standard deviation).

## Data Availability

Data available upon request from the authors.
