# Supplementary material for: Advancing Objective Mobile Device Use Measurement in Children Ages 6–11 Through Built-In Device Sensors: A Proof-of-Concept Study
Source: Hum Behav Emerg Technol. Author manuscript; Available in PMC 2025 Dec 18. (PMC12710754; doi:10.1155/2024/5860114)
Supplement: Supplementary tables and figures [file NIHMS2127363-supplement-Supplementary_tables_and_figures.docx]

**Supplementary Material**

| **Supplementary Table 1. Wearables for Kids (W4K) Protocol** | | | |
| --- | --- | --- | --- |
| **Activity** | **Intensity** | **Description** | **Minutes Spent in Activity** |
| Resting, Lying down | Sed  ≤ 1.5 METs | Lying down in supine position - on a blanket on the ground; playing on iPad (playing games) | 10 |
| Video viewing | Sed  ≤ 1.5 METs | Sitting in a chair at a table playing on iPad (playing games). Instructed to minimize body movements | 5 |
| Walking casual | Light  >1.5 METs to < 3 METs | Walking self-selected pace casual with a research assistant | 5 |
| Walking Brisk | Mod   ≥ 3.0 METs to < 6 METs | Walking self-selected pace brisk with a research assistant | 5 |
| Obstacle course | Vig   ≥ 6.0 METs | Participants will run through an obstacle course led by a research assistant | 5 |
| Break | Sed  ≤ 1.5 METs | Seated research assistant is describing next block | 3 |
| Highlighted portion denotes portion of the protocol used for the current study. | | | |

| **Supplementary Table 2. PATCH Protocol** | | |
| --- | --- | --- |
| **Activity** | **Intensity** | **Minutes Spent in Activity** |
| Supine Resting | 1 – 1.4 METs | 10 |
| Rest (seated) | 1 – 1.4 METs | 5 |
| Stand | 1.5 – 2 METs | 5 |
| Rest (seated) | 1 – 1.4 METs | 5 |
| Walk (light PA) | 2.0 – 3.6 METs | 5 |
| Rest (seated) | 1 – 1.4 METs | 5 |
| Jog/Run – (mod to vid PA) | 5.5 – 10.6 METs | 5 |
| Rest (seated) | 1 – 1.4 METs | 5 |
| Highlighted portion denotes portion of the protocol used for the current study. | | |

| 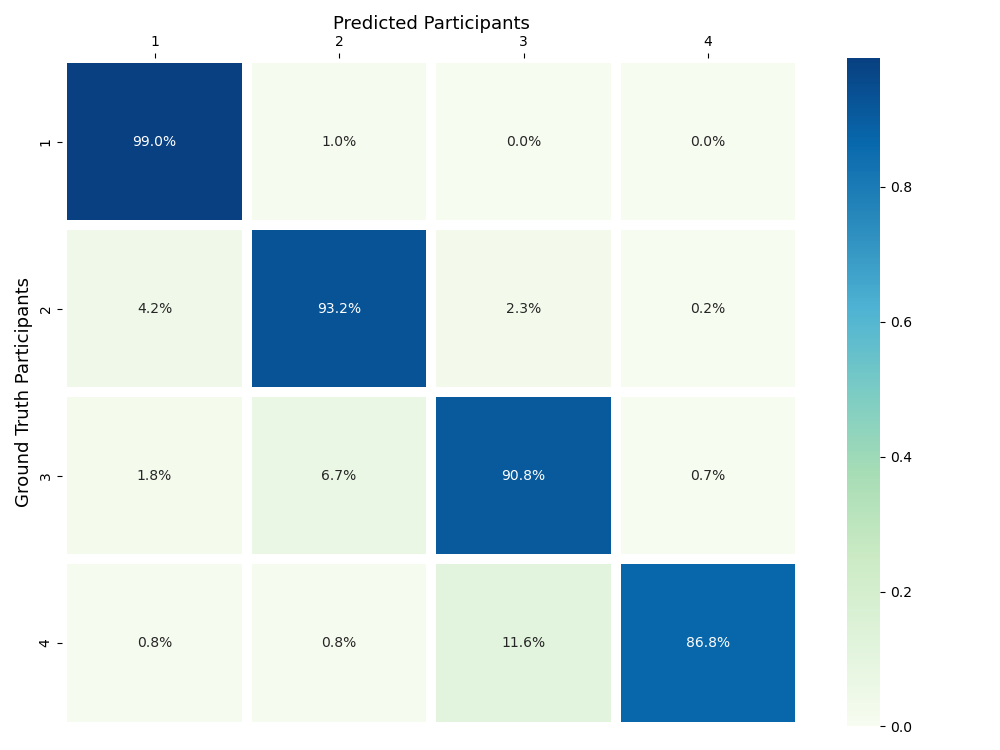  PATCH Random Forest Confusion Matrix | 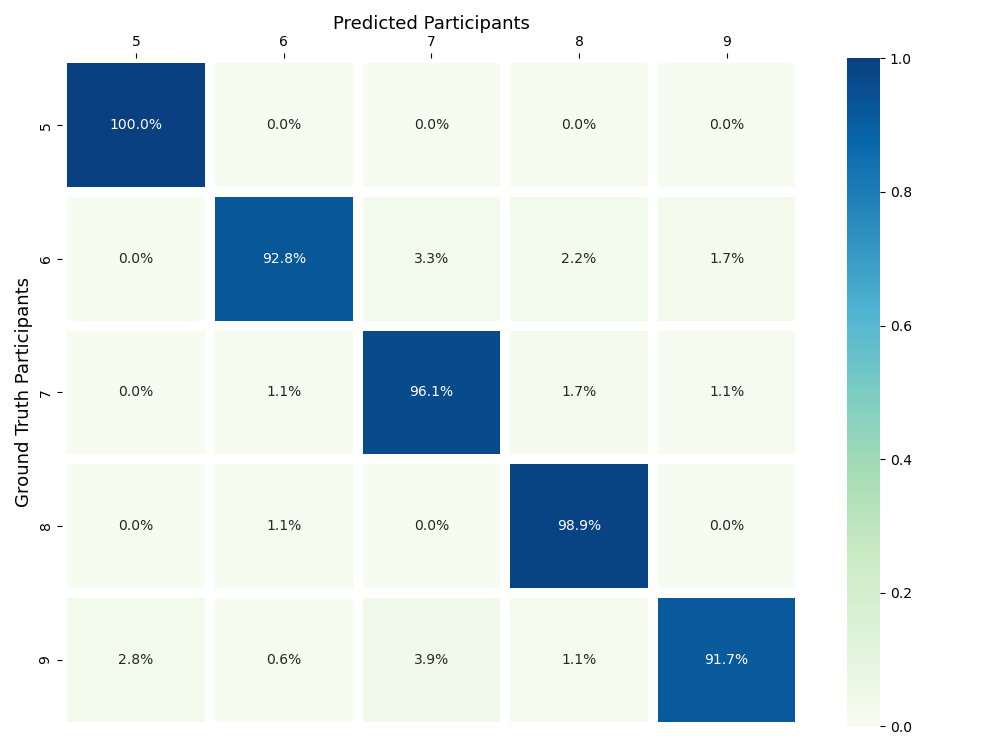  W4K Random Forest Confusion Matrix |
| --- | --- |

**Supplementary Figure 1.** Confusion Matrices of W4K vs. PATCH

| **Supplementary Table 3.** List of All 57 Features |
| --- |
| **Feature Name** |
| Maximum yaw |
| Maximum roll |
| Mean yaw |
| Mean roll |
| Minimum roll |
| Minimum yaw |
| Mean acceleration along Y axis |
| Minimum pitch |
| Maximum acceleration along Y axis |
| Mean acceleration along X axis |
| Mean acceleration along Z axis |
| Maximum acceleration along Z axis |
| Mean vector magnitude |
| Minimum acceleration along Y axis |
| Mean pitch |
| Maximum acceleration along X axis |
| Maximum pitch |
| Minimum acceleration along X axis |
| Root mean square of pitch |
| Minimum acceleration along Z axis |
| Variance of pitch |
| Standard deviation of roll |
| Standard deviation of acceleration along Z axis |
| Root mean square of acceleration along Z axis |
| Variance of acceleration along Z axis |
| Standard deviation of yaw |
| Variance of roll |
| Root mean square of roll |
| Minimum vector magnitude |
| Standard deviation of acceleration along X axis |
| Standard deviation of pitch |
| Maximum vector magnitude |
| Standard deviation of vector magnitude |
| Variance of yaw |
| Kurtosis of acceleration along X axis |
| Skewness of vector magnitude |
| Standard deviation of acceleration along Y axis |
| Root mean square of acceleration along X axis |
| Kurtosis of acceleration along Y axis |
| Root mean square of yaw |
| Root mean square of vector magnitude |
| Root mean square of acceleration along Y axis |
| Variance of vector magnitude |
| Skewness of acceleration along X axis |
| Kurtosis of yaw |
| Variance of acceleration along Y axis |
| Variance of acceleration along X axis |
| Kurtosis of acceleration along Z axis |
| Kurtosis of vector magnitude |
| Skewness of yaw |
| Skewness of roll |
| Kurtosis of roll |
| Skewness of pitch |
| Skewness of acceleration along Y axis |
| Skewness of acceleration along Z axis |

**Supplementary Table 4.** Number of Samples for Training and Testing by Participant

| **Participant** | **Study** | **Training Samples** | **Testing Samples** |
| --- | --- | --- | --- |
| 1 | PATCH | 1676 | 419 |
| 2 | PATCH | 1716 | 429 |
| 3 | PATCH | 1732 | 434 |
| 4 | PATCH | 480 | 121 |
| 5 | W4K | 721 | 181 |
| 6 | W4K | 718 | 180 |
| 7 | W4K | 720 | 181 |
| 8 | W4K | 721 | 181 |
| 9 | W4K | 720 | 180 |


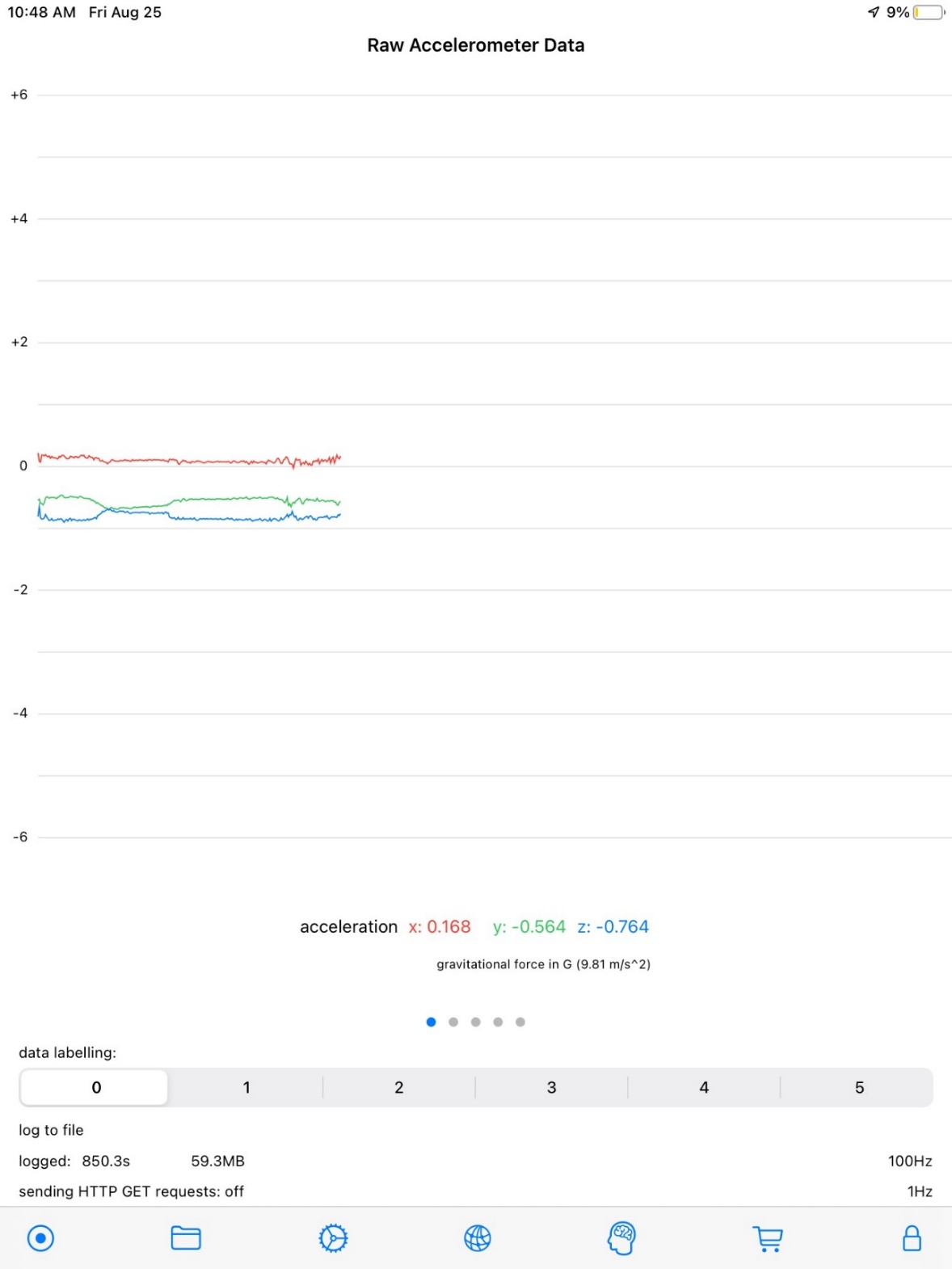


**Supplementary Figure 2.** SensorLog Raw Accelerometer Reading Interface
